# Hemorrhagic Fever with Renal Syndrome in 4 US Soldiers, South Korea, 2005

**DOI:** 10.3201/eid1511.090076

**Published:** 2009-11

**Authors:** Jin-Won Song, Sung-Sil Moon, Se Hun Gu, Ki-Joon Song, Luck Ju Baek, Heung Chul Kim, Todd Kijek, Monica L. O’Guinn, John S. Lee, Michael J. Turell, Terry A. Klein

**Affiliations:** Korea University, Seoul, South Korea (J.-W. Song, S.-S. Moon, S.H. Gu, K.-J. Song, L.J. Baek); 65th Medical Brigade, Seoul (H.C. Kim, T. Kijek, T.A. Klein); US Army Medical Research Institute of Infectious Diseases, Fort Detrick, Maryland, USA (M.L. O’Guinn, J.S. Lee, M.J. Turrell); 1 Current affiliation: Centers for Disease Control and Prevention, Atlanta, Georgia, USA.; 2 Current affiliation: University of North Carolina at Chapel Hill, Chapel Hill, North Carolina, USA.

**Keywords:** Hantavirus, Hantaan virus, hemorrhagic fever renal syndrome, HFRS, Apodemus agrarius, military, South Korea, viruses, dispatch

## Abstract

Four US soldiers acquired hemorrhagic fever with renal syndrome while training near the Demilitarized Zone, South Korea, in 2005. Hantaan virus sequences were amplified by reverse transcription–PCR from patient serum samples and from lung tissues of striped field mice (*Apodemus agrarius*) captured at training sites. Epidemiologic investigations specified the ecology of possible sites of patient infection.

Hantaan virus (HTNV), the etiologic agent for hemorrhagic fever with renal syndrome (HFRS), accounts for ≈70% of all HFRS cases in South Korea and is the most severe of the 4 rodent-borne hantaviruses (Seoul virus, Soochong virus, and Muju virus) found there (*1*–*3*). Recently, a shrew-borne hantavirus, Imjin virus, was isolated from Ussuri white toothed shrews (*Crocidura lasiura*) captured near the Imjin River in South Korea (*4*). The reservoir host of HTNV, the striped field mouse (*Apodemus agrarius)*, is the most abundant field rodent found in South Korea. We conducted an epidemiologic investigation for rodents at 6 training sites near the Demilitarized Zone after 4 US soldiers acquired HFRS in 2005, because no evidence of rodent activity was found where the soldiers worked or resided at their base camp (Camps Hovey and Casey).

## The Study

On October 27, 2005, a US soldier (patient 1) assigned to Camp Hovey, Dongducheon, exhibited signs and symptoms of HFRS (Table 1). The patient was transferred to the Brian Allgood Army Community Hospital (BAACH), Yongsan Army Garrison, Seoul, South Korea, and on October 28, 2005, was confirmed to be seropositive for hantavirus infection by ELISA. A medical advisor initially suspected that the patient may have acquired the infection while sweeping out a dusty storage area at Camp Hovey, where he resided, ≈3 days before the onset of symptoms. A survey of the suspected storage room did not uncover any signs of rodent activity. These data along with the known incubation period of hantaviruses (4–>50 days) prompted a further search for the actual site of transmission.

**Table 1:** History of patients who acquired hantavirus infections while training near the Demilitarized Zone, South Korea, during 2005*†

Patient no.	Onset‡	TMC report	Date of diagnosis§	Date confirmed¶	Ribavirin therapy	Date discharged	Training dates (all locations)	Incubation period, d	Training dates (infection source)	Estimated incubation period, d
1	Oct 25	Oct 27	Oct 28	Nov 1	Oct 30	Nov 5	Sep 20–29	26–35	Sep 25–29 (FP-60)	26–30
2	Nov 3	Nov 8	Nov 9	Nov 12	Nov 9	Dec 8	Oct 8–21	13–26	Oct 8–18 (TBTA-S)	16–26
3	Nov 5	Nov 9	Nov 12	Nov 16	Nov 13	Nov 20	Oct 8–15	21–28	Oct 8–15 (TBNA-N)	21–28
4	Nov 12	1st, Nov 13; 2nd, Nov 14	Nov 15	Nov 17	No	Nov 20	Oct 8–21	22–35	Oct 8–18 (TBTA-S)	25–35

*TMC, Troop Medical Clinic; HFRS, hemorrhagic fever renal syndrome; FP, firing point; TBTA, Twin Bridges Training Area; S, south; N, north. †HFRS type was Hantaan virus for all infections. Patients 1 and 3 were hospitalized for 8 days, patient 4 for 5 days, and patient 2 for 29 days. Patient 2 was sent to Samsung Hospital for dialysis during part of his hospitalization at Brian Allgood Army Community Hospital (BAACH). US Army Medical Department–Korea does not have dialysis capability. ‡Onset of symptoms. §Diagnosis by ELISA at BAACH. ¶Confirmation of hantavirus type by reverse transcription–PCR, Korea University.

**Figure 1:**
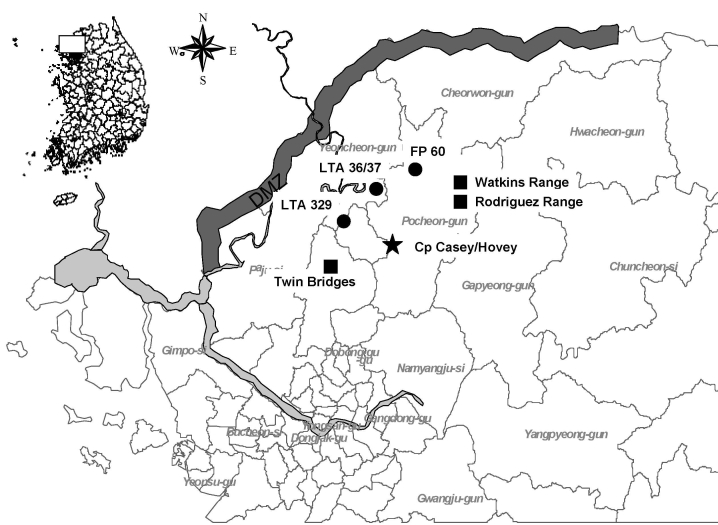
Location of training sites where hemorrhagic fever with renal syndrome (HFRS) patients 1–4 conducted training exercises 50 days before the onset of illness. Rodent surveillance was not conducted at Watkins Range due to limited exposure. DMZ, Demilitarized Zone; LTA, local training area; solid circles, military training sites of patient 1; solid squares, military training sites of patients 2, 3, 4; star, base camp.

A blood sample from patient 1, tested by both the indirect immunofluorescent antibody (IFA) technique and reverse transcription–PCR (RT-PCR), confirmed that the patient was infected with HTNV. An epidemiologic survey was conducted, and results showed that the patient had trained at 4 US- and South Korea–operated training sites from 26 to 35 days before the onset of symptoms. The patient had first trained at local training area (LTA) 320, then at LTA 36/37, and finally at firing point (FP)-60 (Figure 1). The training consisted of setting up firing positions, establishing a cantonment site, and performing other training activities for 5 days before moving on to FP-60, where troops conducted firing exercises from September 25–29, 2005, before returning to Camp Hovey.

On November 8–9, 2005, two soldiers (patients 2 and 3) from Camp Casey, Dongducheon, had signs and symptoms of HFRS and so were sent to the BAACH where they received a diagnosis of HFRS (Table 1). Patient 4 from the same unit sought treatment at the Camp Casey Troop Medical Clinic on November 13 with low-grade fever, decreased appetite, abdominal pain, chills, low back pain, nausea, and vomiting, and was provided fluids and assigned to quarters (home bed rest). On November 14, laboratory results indicated a nonspecific proteinuria characteristic of HFRS infections, and the patient was transported to the BAACH, where HFRS was diagnosed. Blood samples from all 3 patients were positive for HTNV by IFA and RT-PCR. All 3 patients had trained at Twin Bridges Training Area (TBTA) with potential incubation periods that ranged from 16 to 35 days, and 2 (patients 2 and 4) had trained together at Rodriguez and Watkins Ranges 3 days before conducting a tactical move to TBTA (Table 1; Figure 1). Patients 2 and 4 conducted training at TBTA-North (N) and TBTA-South (S), and patient 3 only conducted training at TBTA-N.

Small mammal trapping was conducted at US and South Korea–operated training sites where the HFRS patients had previously trained within 60 days. Preseasonal (September) *A. agrarius* mice trapping rates were relatively low at both FP-60 (11.4%) and Rodriguez Range (7.6%), and although postseasonal trapping rates were high at FP-60 (42.3%), they remained relatively low at Rodriguez Range (12.1%) (Table 2). During the fall, hantavirus seropositive rates were high at FP-60 (20.0%) and Rodriguez Range (31.3%). During the winter, seropositive rates increased at FP-60 (25.8%), but decreased at Rodriguez Range (15.7%). During the winter, seropositive rates at TBTA-N (26.3%) and TBTA-S (37.8%) were high, but were relatively low at other training sites (LTA320/36/37) surveyed.

**Table 2:** Results of rodent-borne disease surveillance at FP 60 and LTAs 36, 37, and 320, Rodriguez Range and Twin Bridges Training Area (South and North Bowls), Gyeonggi Province, South Korea, 2005*

Location	Spring			Summer			Fall			Winter	
	Trapping rate† (%)	Seropositive rate (%)		Trapping rate (%)	Seropositive rate (%)		Trapping rate (%)	Seropositive rate (%)		Trapping rate (%)	Seropositive rate (%)
FP 60	21/220 (9.5)	2/21 (9.5)		89/220 (40.5)	12/89 (13.5)		25/220 (11.4)	5/25 (20.0)		93/220 (42.3)	24/93 (25.8)
LTA 36/37	ND	ND		ND	ND		ND	ND		23/90 (25.6)	0/23 (0)
LTA 320	ND	ND		ND	ND		ND	ND		26/90 (28.9)	2/26 (7.7)
Rodriguez	38/180 (21.1)	3/38 (7.9)		29/210 (13.8)	7/29 (24.1)		16/210 (7.6)	5/16 (31.3)		33/210 (15.7)	4/33 (12.1)
TBTA-N	ND	ND		ND	ND		ND	ND		19/180 (10.6)	5/19 (26.3)
TBTA-S	ND	ND		ND	ND		ND	ND		45/180 (25.0)	17/45 (37.8)

*FP, firing point; LTAs, local training area; ND, not determined; TBTA, Twin Bridges Training Area; N, north; S, south. †Trapping rate, total number of rodents trapped/number of traps.

Blood samples from each of the 4 patients and lung tissues of seropositive rodents were assayed by RT-PCR. RNA extracted by using RNA-Bee isolation kit (TEL-TEST Inc., Friendswood, TX, USA) was reverse transcribed by using the superscript II RNase H-reverse transcriptase kit (GIBCO-BRL, Gaithersburg, MD, USA). Primers (outer primer set, 5′-TGGGCTGCAAGTGC-3′, 5′-ACATGC TGTACAGCCTGTGCC-3′; inner primer set, 5′-TGGGCTGCAAGTGCATCAGAG-3′, 5′-ATGGATTACAACCCCAGCTCG-3′) amplified a 373-nt region of the hantavirus G2-encoding medium (M) segment (*1*,*5*,*6*). Amplified products were fractionated according to size by electrophoresis on 1.5% agarose gels containing ethidium bromide (0.5 mg/mL). DNA sequencing was performed in both directions, by using the dye primer cycle sequencing ready reaction kit (Applied Biosystems, Foster City, CA, USA) on an automated sequencer (Model 3730XL; Applied Biosystems).

**Figure 2:**
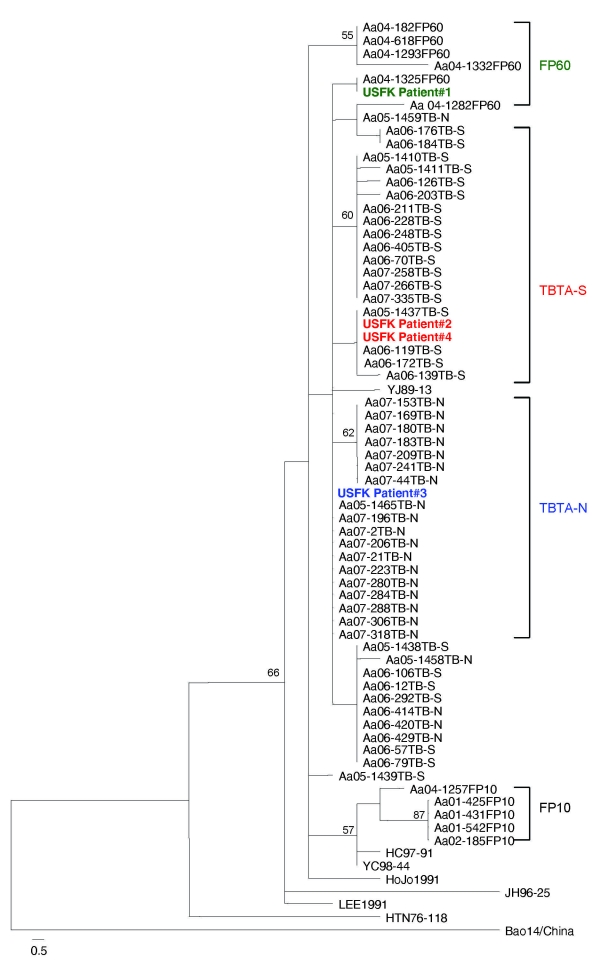
Phylogenetic tree by maximum parsimony method, rooted at the midpoint, based on the 320-bp region of G2 glycoprotein–encoding medium segment of 4 hemorrhagic fever with renal syndrome patients who were US soldiers in South Korea (patients 1–4), 2005 (GenBank accession nos. FJ561275–FJ561278) and field mice (*Apodemus* spp*.*)–borne Hantaan viruses (HTNV). HTNV sequence amplified from patient 1 was identical with a HTNV sequence (Aa04–1325) from *A. agrarius* mice captured at firing point (FP) 60. HTNV sequences from patients 2 and 4 were the same as 3 HTNV sequences (Aa05–1437, Aa06–119, Aa06–171) from *A. agrarius* mice captured at Twin Bridge Training Area–South (TBTA-S), and the HTNV sequence from patient 3 was identical to 11 HTNV sequences (Aa05–1465, Aa07–2, Aa07–21, Aa07–196, Aa07–206, Aa07–223, Aa07–280, Aa07–284, Aa07–288, Aa07–306 and Aa07–318) from *A. agrarius* mice at Twin Bridge Training Area-North (TBTA-N). Branch lengths are proportional to the number of nucleotide substitutions, while vertical distances are for clarity only. The numbers at each node are bootstrap probabilities (expressed as percentages), as determined for 100 iterations by using PAUP version 4.0b (http://paup.csit.fsu.poly). The colors indicate patients and corresponding training sites.

Phylogenetic analysis, both neighbor-joining and maximum-parsimony methods, based on the 320-nt region of the G2 glycoprotein–encoding M segment of the 4 HFRS patients, and HTNV sequences amplified from *A. agrarius* mice captured at FP-10, FP-60, TBTA-N, and TBTA-S demonstrated that the HTNV sequence amplified from patient 1 was identical to sequences from the *A. agrarius* HTNV strain (04–1325) at FP-60. The analyses also demonstrated that the HTNV sequences from patients 2 and 4 were identical to *A. agrarius* HTNV sequence (05–1437) at TBTA-S, and the HTNV sequence from patient 3 was identical to *A. agrarius* HTNV sequences (07–196 and 05–1465) at TBTA-N. These data demonstrate the most likely site of infection for the 4 HFRS patients (Figure 2).

## Conclusions

The relationships between rodent density, the proportion of hantavirus-seropositive rodents, and incidence of human infection are complex and poorly understood (*7*,*8*). Previous literature indicates that a prevalence of hantavirus seropositivity >20% among *A. agrarius* mice greatly increased the risk for transmission (*9*–*11*). Although monitoring rodent populations may provide some warning, the most effective means of controlling hantavirus infections is limiting human contact with rodents and the inhalation of dust with virus-laden rodent excreta (*12*).

The large patches of tall dense grasses narrowly separated by barren ground where artillery firing is conducted at FP-60 provide harborage for *A. agrarius* mice and, during the winter trapping period, yielded ≈20% capture rates (*10*). Eliminating these large grassy islands, and thus the rodents that inhabit these areas, and cutting the tall grasses and scrub vegetation to <10 cm along the training site perimeter would decrease hantavirus infection risks by reducing the rodent populations. Efforts to mitigate disease risks through modernization of training sites, cutting of vegetation that increases predation of small mammals, and habitat reduction within 50 m of military operations, where possible, are being instituted at selected US-operated training sites as a result of consolidation and modernization of large, multipurpose range complexes.

Previously, phylogenetic analysis of a 324-nt region of the G2 glycoprotein-encoding M genomic segment has been shown to be representative of the entire M segment (*6*). This region may be useful for classifying newly identified hantaviruses (when cross-neutralization data are unavailable) or for further analysis of molecular phylogeny of hantaviruses spatially. The genome of HTNV sequences obtained from patient 1 was identical to a viral sequence from an *A. agrarius* field mouse captured at FP-60 and those from patients 2 and 4 were identical to 3 viral sequences from *A. agrarius* at TBTA-South, and patient 3 was identical to 11 viral sequences from *A. agrarius* field mice at TBTA-North. These data showed the epidemiologic link between US soldier patients and rodent hosts at the training sites near the Demilitarized Zone in South Korea.
